# Mechatronic Prototype of Parabolic Solar Tracker

**DOI:** 10.3390/s16060882

**Published:** 2016-06-15

**Authors:** Carlos Morón, Jorge Pablo Díaz, Daniel Ferrández, Mari Paz Ramos

**Affiliations:** 1 Sensors and Actuators Group, Department of Construction Technology, Polytechnic University of Madrid, Madrid 28040, Spain; daniel.ferrandez.vega@alumnos.upm.es; 2 Professional Institution Salesiana, Salesianos Carabanchel, Madrid 28044, Spain; jdiaz@salesianoscarabanchel.com (J.P.D.); pramos@salesianoscarabanchel.com (M.P.R.)

**Keywords:** energetic simulation, concentrating solar thermal power, architectural integration, mechatronic solar tracker, parabolic, infrared thermography

## Abstract

In the last 30 years numerous attempts have been made to improve the efficiency of the parabolic collectors in the electric power production, although most of the studies have focused on the industrial production of thermoelectric power. This research focuses on the application of this concentrating solar thermal power in the unexplored field of building construction. To that end, a mechatronic prototype of a hybrid paraboloidal and cylindrical-parabolic tracker based on the Arduido technology has been designed. The prototype is able to measure meteorological data autonomously in order to quantify the energy potential of any location. In this way, it is possible to reliably model real commercial equipment behavior before its deployment in buildings and single family houses.

## 1. Introduction

In recent decades, the increasing demand for energy has encouraged the development of new renewable energy production systems with more competitive economic costs and lower environmental impact [1]. Solar power is one of the clean energies able to reduce human need for fossil fuel consumption. One of the main uses of solar power is electricity production based on two existing on the market technologies: photovoltaic panels and concentrating collectors [2,3]. The former are used for the production of energy from both direct and diffuse radiation, working at moderate temperatures often under 100 °C: therefore they are less dependent on exhaustive solar tracking. On the other hand, the latter function requiring concentrating strategies and high temperatures, using in most cases direct solar radiation and requiring complete solar tracking.

**Figure 1:**
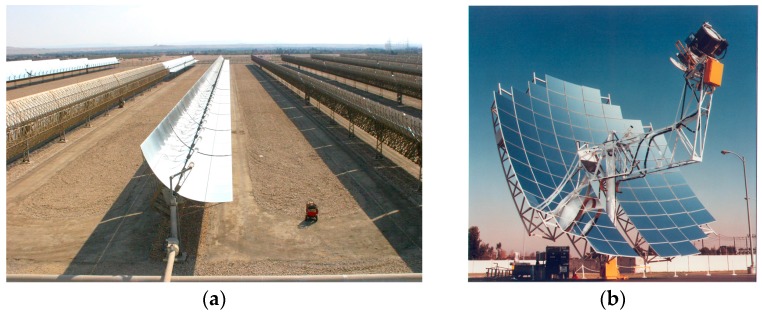
Examples of solar thermal plants: (**a**) Parabolic-trough collector technology; (**b**) Paraboloidal solar collector with Stirling engine. Source: Flickr Creative Commons.

This research work is focused on the second capturing technology, based specifically on cylindrical-parabolic and paraboloidal concentrating for electricity production by the Rankine and Stirling thermodynamic cycles, which are considered to be highly efficient and productive methods [4,5,6]. Currently this type of collectors are widely applied at the industrial level (Figure 1), to produce large amounts of energy in solar thermal power plants [7,8]. That is why the idea to integrate this type of systems in the building sector is remarkable, taking into consideration its high yields.

Some authors have tried to improve architectural integration of these systems [9,10], making them more flexible and esthetic for use of space. All of these authors agree on the need to position the collector correctly in order to achieve performance similar to that obtained under laboratory conditions. It is for this reason that solar tracking systems play a crucial role in the development of solar thermal energy systems, making it possible to state that collector efficiency directly depends on the quality of tracking equipment which can constantly provide the maximum amount of direct solar radiation [11,12].

Furthermore, the solar tracking mechanism has to be able to track the Sun´s movement with a high level of precision, returning the collector to its original or at-rest position at the end of the day or in unfavorable weather conditions, even performing optimal tracking on cloudy days. In this regard, there are two types of solar trackers: those which use solutions based on astronomical ephemerides with open-loop control, and those which are based on active tracking systems with close-loop approximations [13].

In the first case, the tracking is performed following satellite-based estimates obtained through incoming radiation reflection on the Earth. On occasion, to this source of information the possibility to include historical and empirical data of visible and infrared spectrum obtained using weather stations placed on the Earth’s surface is added. Finally, this set of data is calculated mathematically using interpolation algorithms between direct and global radiation [14]. The problem resides in the fact that the resolution obtained using this method (3 km × 3 km) is not always optimal. Moreover, obtained data is horizontal what requires one to introduce inclination angle correction factors. The statistical process in turn gives inevitable errors when working with large database compounding the problem of sensor degradation and saturation.

The trackers which are able to interpret direct solar radiation received through the use of sensors belong to the second type [15]. These photosensitive sensors are used to continually send a signal to data processing equipment, which allows real-time continuous tracking of the Sun during the whole day.

The most commonly used tracking configuration is the one which is able to perform at the same time azimuth and zenith (or polar) movements of the collector [16]. For the azimuth tracking the tracker has to rotate from east to west following parallel to the Earth’s surface trajectory. Instead, for the zenith or raising movement solar tracker has to rise following the solar trajectory from sunrise, and descend slowly to sunset varying its height in different seasons of the year. Both movements should be carefully coordinated and performed as accurately as possible. However, there are less precise solar trackers which use only azimuth movement presetting determined inclination angle depending on area latitude.

The solution presented in this research is based on the fabrication of mechatronic solar thermal tracker with hybrid, paraboloidal and cylindrical-parabolic technology, in such a way that it is possible to capture data of different measurable physical magnitudes, and at the same time to simulate two axis tracking of both technologies. For the construction of this prototype, it was necessary to carry out three-dimensional design and simulations using computer software which allowed us to validate a solar tracker prototype. Subsequently, the data was collected *in situ* in order to improve and calibrate the mechatronic tracker. Finally, with a view to improve the architectural integration of this type of energy technologies in the building sector, the work was focused on the paraboloidal disc technology in combination with the Stirling cycle as the most promising solution for possible deployment in buildings.

## 2. Materials and Methods

In this section the tasks related to design and manufacturing of the CSP tracker are described.

### 2.1. Design of the 3D Prototype

**Figure 2:**
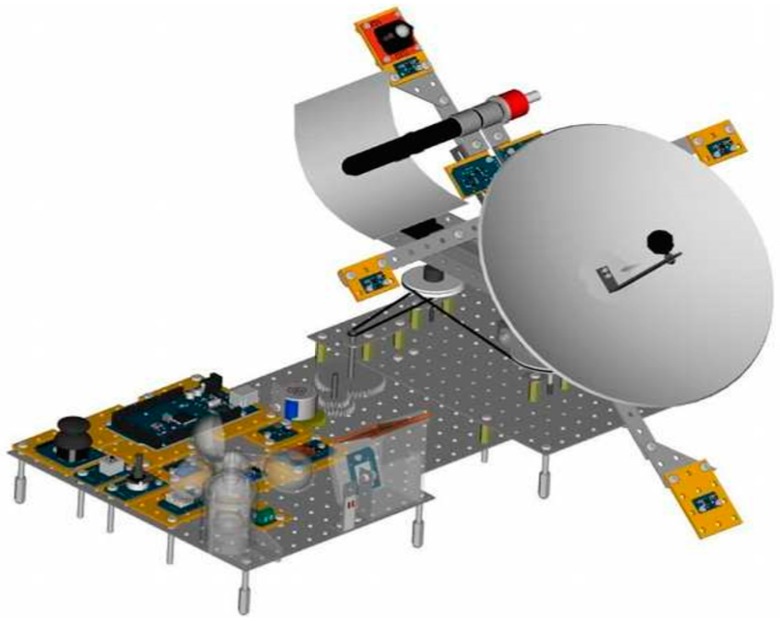
3D design of the CSP tracker prototype.

The mechanical, optical and electronic 3D design was made with a powerful CAD software known as FreeCAD 0.15 [17], which allows one to give parameters to all the components of the drawing, and is compatible with other standard design formats. Figure 2 shows fully designed CSP solar tracker.

Without describing in detail the 3D design process, but focusing rather on the components and devices that define it, it is possible to establish the following classification of subsystems:

- Mechanical subsystem: made up of the aluminium framing, plastic fasteners, stainless steel screws, gears and torque transmission belts, bolts and sleeves.
- Optical subsystem: formed by one paraboloidal collector plus a parabolic trough, being both of them made of highly reflective aluminium.
- Electronic subsystem: constituted with different physical magnitudes sensors (pressure, temperature, humidity, wind, irradiance, presence of water and air quality); positioning variables (accelerometer, compass and camera); driving devices (step by step motor and linear actuator); Arduino programmable electronic controller and many other additional devices [18,19].

The most representative components that define the device are listed in Table 1.

### 2.2. Definitive State of Mechatronic Solar Tracker

**Figure 3:**
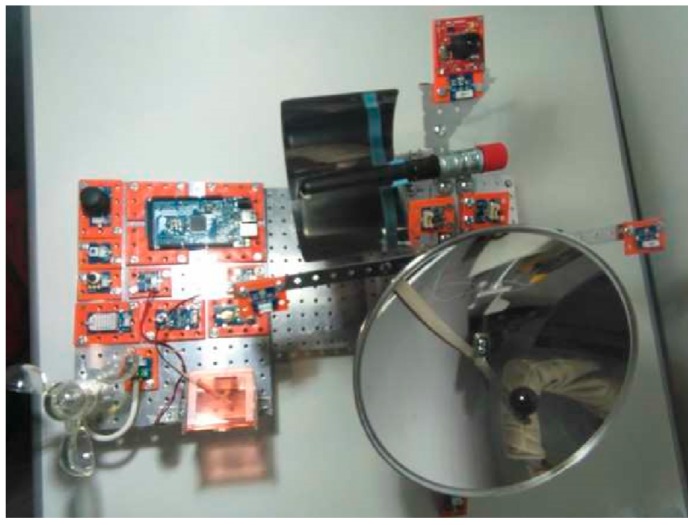
Final state of the solar thermal tracker.

After the previous design tasks, the next step was the real construction of the mechatronic prototype. The definitive result can be shown in Figure 3, with all the sensors of the CSP tracker already installed plus the driving devices and solar collectors. This solar tracker prototype can work in real irradiance exposed to outdoors conditions, on the situation that these meteorological conditions allow to do so, because it does not have encapsulation for any waterproof sensor. But it is true that its behaviour can be analyzed by mean on controlled tests in laboratory, or even with computing dynamic simulators that model atmospheric conditions.

### 2.3. Tracking System Design and Arrangement

**Figure 4:**
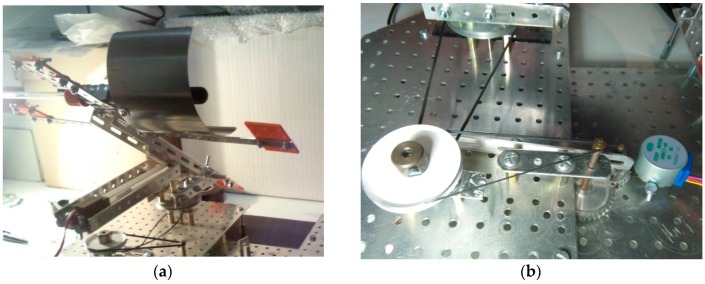
Details of the two axis tracking mechanism. (**a**) Linear actuator; (**b**) Stepper motor.

In order to show in detail the two axis tracking mechanism of the prototype, it can be useful to have a close look to the linear actuator and stepper motor included. In relation to the first one, as Figure 4a shows, the linear actuator is responsible for the tilt movement of the tracker, since it has an axial rod with a tiny but powerful internal gearbox and a rectangular section to increase its rigidity. Its working principle consists in pulling or pushing a certain load (solar collectors in this case) throughout the length of the rod. The speed of displacement depends on the gearbox mentioned before and the load the actuator is facing.

In the same way, the stepper motor is the device that describes the azimuth movement in the best way: to position in a reliable and precise way the azimuth of the tracker Figure 4b, together with a torque enough to move the load (solar collectors again). Specifically, the shaft of the stepper has to move a gear assembly that moves in its turn another shaft that impels two belt pulleys engaged.

### 2.4. Definition of the Solar Tracking Algorithm

**Figure 5:**
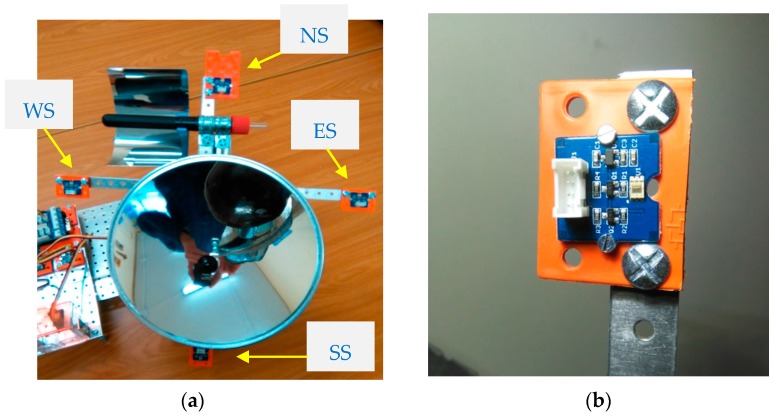
Data capture equipment. (**a**) Position of four irradiance sensors; (**b**) Detail of an irradiance sensor.

With an aim of describing better how the prototype tracks the Sun rays, it is also important to indicate how the irradiance sensors work. Firstly, it is necessary to take into account that there are four sensors located in cross in positions North-South-Est-West, which mission is to capture irradiance constantly (Figure 5). 

According to the values of irradiance of each sensor and the algorithm described later (seeking the maximum perpendicularity to sun rays, a mandatory condition to concentrate them in both collectors), the Arduino controller activates linear actuator or stepper motor shown before. Electronically, this kind of sensor is based on an irradiance to digital signal converter with the capability to measure a wide range of radiance spectrum using built-in dual sensibility diodes. In order to face the acquisition of irradiance data, it is possible to select between three detection modes: infrared mode (especially interesting for CSP); full spectrum; and visible spectrum for human being. In terms of irradiance and luminosity (respectively and making a conversion between them) the working range includes from 0 W/m^2^ (0.1 lux) up to 1000 W/m^2^ (40,000 lux).

Subsequently, an algorithm for two axis solar tracking was developed. To this end, it was necessary to identify the most important tasks and to modernize them. There are two operations: azimuthal movement (East-West) and zenithal movement (North-South).

Firstly, it was necessary to identify by abbreviations the names of the irradiance sensors, which intervene in the tracking algorithm, as it is shown in Table 2.

**Figure 6:**
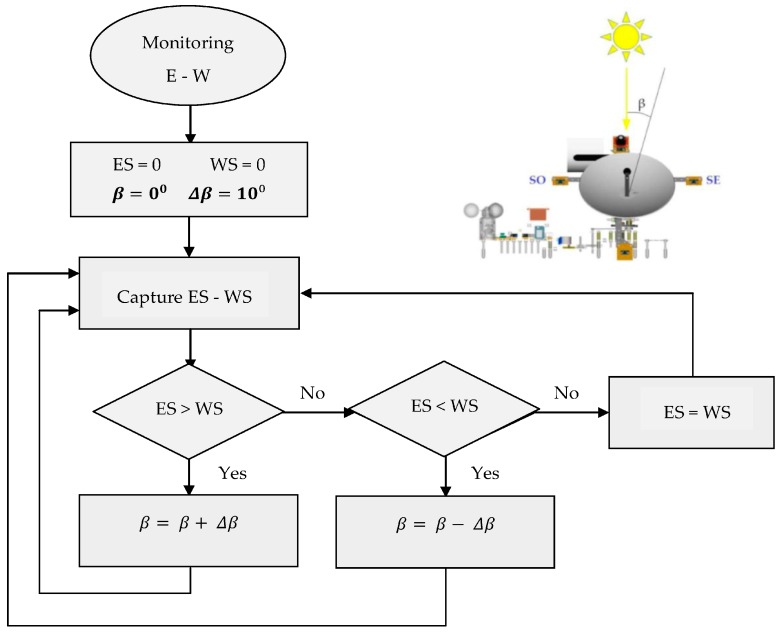
Azimuthal tracking flow chart.

The azimuthal (East-West) tracking works according to the principle shown in the flow chart of Figure 6. At first, the variables are reset, where the azimuthal angle β can be increased in steps of Δβ=10° (user-adjustable value). Once the controller captures data from ES and WS sensors, if any of them is greater than the other one, this will imply a higher irradiance level in itself, and therefore, it will be necessary to increase or decrease that azimuth β in steps of Δβ searching for the maximum perpendicularity with respect to the Sun, ensuring the highest solar irradiation.

**Figure 7:**
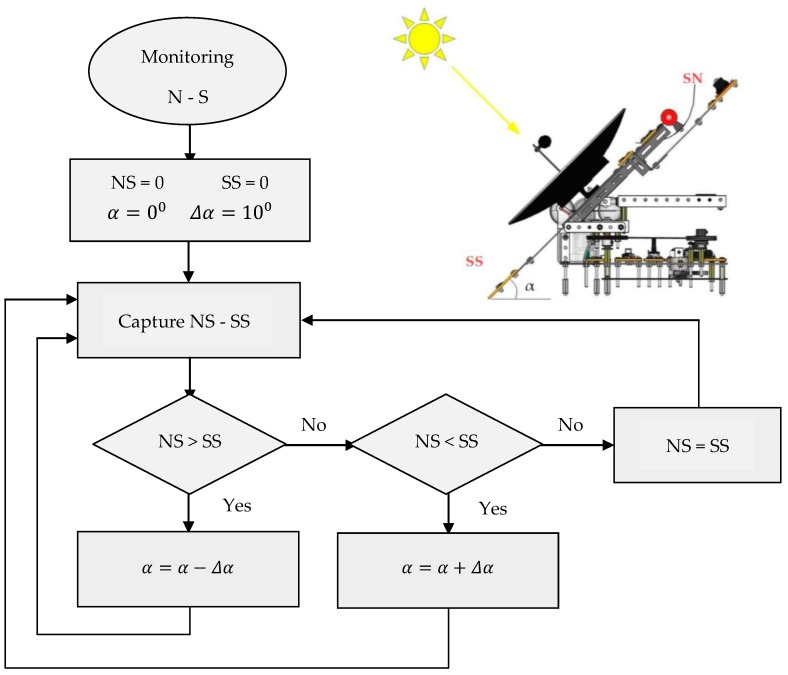
Elevation tracking flow chart.

In the same way, the elevation (North-South) tracking can be done, where its working principle is shown with the help of the flow chart of Figure 7. As mentioned before, the variables are reset, where the elevation angle α can be increased in steps of Δα=10° (user-adjustable value too). Once the controller captures data from NS and SS sensors, it detects oscillations in irradiance values, if any of them is greater than the other one. So, the mechatronic device is able to increase or decrease that elevation α in steps of Δα searching for the maximum perpendicularity with respect to the sun, achieving the higher solar radiation.

Combining these two movements (azimuthal and elevation), the optimal position that allows to gain the maximum use of solar rays can be obtained, which therefore, permits to obtain a higher performance of the solar prototype. Because of the simplicity of Arduino-compatible devices, the programming of this controller is easy if the sequences detailed are followed as shown in the flow charts.

On the other hand, it can be useful to model the tracking system of the prototype, by means of Laplace Transforms. These automatic control systems can command the variables that characterize any physical process and are built with the following items: input signal–process–output signal. The first one (input signal) is also known as control signal, set point or reference, and is used to command the process itself. And finally, the output signal is nothing but the variable to control.

The open-loop control system (non-feedback controller) computes its input into a system using only the current state and its model of the system. In this sense, a characteristic of this type of controller is that it does not use feedback to determine if its output has achieved the desired goal of the input. In such a way, the system does not observe the output of the processes that it is controlling. Consequently, an open-loop system cannot correct any errors that it could make, and that is why it also may not compensate disturbances in the system, what is not valid for the solar trackers.

On the contrary, the close-loop control system (feedback controller) is capable of using a feedback loop to control the behavior of any system by comparing its output with respect to a desired value with the help of a sensor. This way, is possible to calculate the difference as an error signal to dynamically change the output. Obviously, it is closer to the desired output and it is the strategy used in this paper as it can be seen below.

**Figure 8:**
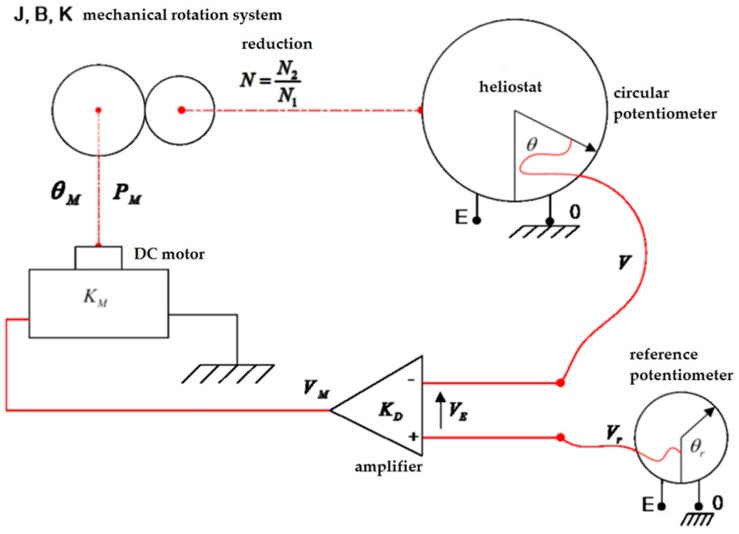
Modelling of the solar tracker in terms of differential equations.

In effect, once the features of all the techniques related to automatic control are seen, it is interesting to obtain the Transference Function that models the working principles of a solar tracker. To do so, firstly the differential equations of the physical model are considered in terms of variable time (t) according to the model as it is shown in Figure 8.

(1) $$v\left(t\right)=\frac{E}{2\pi }\cdot \theta \left(t\right)$$

(2) $$v_{r}\left(t\right)=\frac{E}{2\pi }\cdot \theta_{r}\left(t\right)$$

(3) $$v_{E}\left(t\right)=v_{r}\left(t\right)-v\left(t\right)$$

(4) $$v_{M}\left(t\right)=\;K_{D}\cdot v_{E}\left(t\right)$$

(5) $$p_{M}\left(t\right)=K_{M}\cdot v_{M}\left(t\right)$$

(6) $$p_{M}\left(t\right)=J\cdot \frac{d^{2}\theta_{M}\left(t\right)}{dt^{2}}+B\cdot \frac{d\theta_{M}\left(t\right)}{dt}+K\cdot \theta_{M}\left(t\right)$$

(7) $$\frac{N_{1}}{N_{2}}=\;\frac{\theta_{2}\left(t\right)}{\theta_{1}\left(t\right)}$$

where θ(t) is the angular position of the tracker, $\theta_{r}\left(t\right)\;$is the reference angular of the tracker, v(t) is the voltage obtained, $v_{r}\left(t\right)\;$is the reference voltage from the potentiometer, between 0 V up to EV, $v_{E}\left(t\right)\;$is the diference between $v_{r}\left(t\right)$ and v(t), $K_{D}\;$is the gain of the amplificatory, $p_{M}$ is the torque of the DC motor, $K_{M}\;$is the proportionality constant of the DC motor, J is the inertia of the load, B is the friction between joints, K is the elastic torque, $\theta_{M}\left(t\right)\;$is the angle moved by the tracker and N is the gear relation.

Therefore, once the Laplace Transform is aplied to the expressions mentiond before, making a change of variable in terms of the new variable s = jw, and acording to its mathematical definition, the following set of aditional equations can be obtained:

(8) $$L\;\left[f\left(t\right)\right]=F\left(s\right)=\overset{\infty }{\underset{0}{\int }}e^{-t\cdot s}\cdot f\left(t\right)\cdot dt$$

(9) $$V\left(s\right)=\frac{E}{2\pi }\cdot \theta \left(s\right)$$

(10) $$V_{r}\left(s\right)=\frac{E}{2\pi }\cdot \theta_{r}\left(s\right)$$

(11) $$V_{E}\left(s\right)=V_{r}\left(s\right)-V\left(s\right)$$

(12) $$V_{M}\left(s\right)=\;K_{D}\cdot V_{E}\left(s\right)$$

(13) $$P_{M}\left(s\right)=K_{M}\cdot V_{M}\left(s\right)$$

(14) $$P_{M}\left(s\right)=\left(J\cdot s^{2}+B\cdot s+K\right)\cdot \theta_{M}\left(s\right)$$

(15) $$\frac{N_{1}}{N_{2}}=\;\frac{\theta_{2}\left(s\right)}{\theta_{1}\left(s\right)}$$

**Figure 9:**
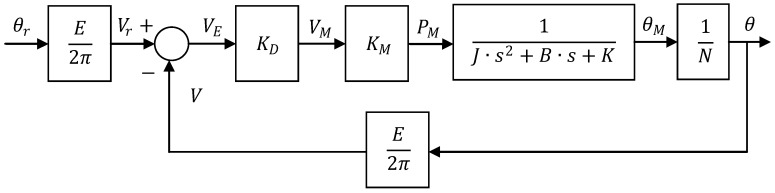
Close-loop block diagram for one axis of the solar tracker.

Figure 9 shows the close loop working block diagram of the solar tracker in terms of transfer function.

Even though this mathematical tool is really powerful, trying to simulate ‘by hand’ a device such as the solar tracker in order to predict its behaviour against wind, increase/decrease of irradiance and so on, is a very tedious task. However, it can be very useful to apply this model with the use of simulation environment software such as Modelica, Wolfram Systemodeller or Simulink.

### 2.5. Energetic Characterization

Before modelling the previous working tasks of the solar tracker, it was energetically characterized according to real measurements of paraboloidal and parabolic trough collector. Even though this prototype is not going to produce energy, it can be interesting to make this energetic characterization in order to foresee results for commercial collectors. In order to achieve this, Greenius 4.1.1 [17] software with meteorological data from the latest years in Madrid can be used.

In relation to the paraboloidal collector, the equations that model the power generated are:

(16) $$P_{gro}=a\cdot E_{corr}+b\;\Leftrightarrow \;E_{DNI}\;\geq E_{DNI,\;min}$$

(17) $$P_{gro}=0\;\Leftrightarrow \;E_{DNI}<E_{DNI,\;min}$$

where $P_{gro}$ is the gross electrical power generated in W, $E_{corr}$ is the corrected irradiance in W/m^2^ that can be obtained in Equation (18), $E_{DNI}$ is the direct normal irradiance in W/m^2^, $E_{DNI,\;min}$ is the minimum $E_{DNI}$ to start the production in W/m^2^, *a* and *b* are two performance constants from the model, in W_e_/(W/m^2^). In this way:

(18) $$E_{corr}=E_{DNI}\cdot f_{ref}\cdot f_{tem}$$

where $f_{ref}$ and $f_{tem}$ are the reflection and temperature correction factors obtained by:

(19) $$f_{ref}=f_{sha}\cdot f_{int}$$

Here $f_{sha}$ is the shadowing factor and $f_{int}$ is the interception factor. So:

(20) $$f_{tem}=\frac{\theta_{nor,amb}+273,15℃}{\theta_{amb}+f_{ref}\cdot E_{DNI}\cdot c_{coo}+273,15℃}$$

where $\theta_{nor,amb}$ is the normalized temperature for the performance of the model in ℃, $\theta_{amb}$ is the higher temperature reachable in Stirling thermodynamic cycle in ℃ and $c_{coo}$ is the cooling constant in ℃/(W/m^2^).

On the other hand we also considered the parasitic power with the help of:

(21) $$P_{par}=P_{ope}\;\Leftrightarrow \;E_{DNI}\geq E_{DNI,\;min}$$

(22) $$P_{par}=P_{sle}\;\Leftrightarrow \;E_{DNI}<E_{DNI,\;min}$$

where $P_{par}$ is the parasitic global power in W, $P_{ope}$ is the operating power in W and $P_{sle}$ is the sleeping power in W.

In case of power injected to net, it can be obtained with the Equation (8):

(23) $$P_{gri}=P_{gro}-P_{par}$$

where $P_{gri}$ is the injected power towards net or consumed from net in W.

**Figure 10:**
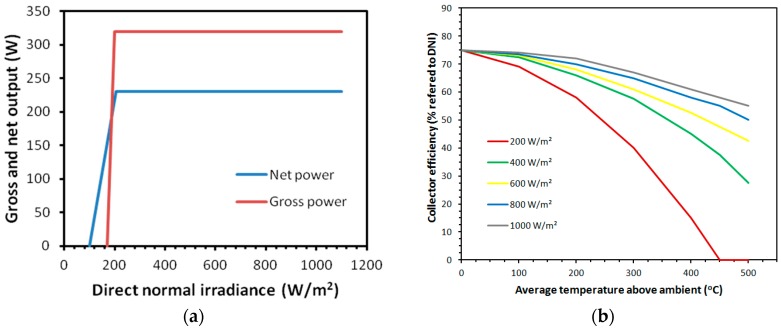
Energetic characterization of collectors. (**a**) Energetic profit obtained by the paraboloidal collector; (**b**) Energetic performance of parabolic trough collector.

The gross profit in energy obtained for our paraboloidal collector using equations shown before, can be seen in Figure 10a. In the same way, for the case of cylindrical-parabolic collector a modelling process was designed according to the following equations that allow to obtain its performance:

(24) $$\eta_{col}=K\cdot \eta_{opt,\;0}\cdot \eta_{cle}-\left(K\cdot b_{0}\cdot \Delta T+\frac{b_{1}\cdot \Delta T+b_{2}\cdot \Delta T^{2}+b_{3}\cdot \Delta T^{3}+b_{4}\cdot \Delta T^{4}}{DNI}\right)$$

where $\eta_{col}$ is the performance of the cylindrical-parabolic collector, $b_{i}\;\left(i=0-4\right)$ are the heat losses in vacuum tubes estimated experimentally, $\eta_{opt,\;0}$ is the optical performance for the collector for θ=0°, θ is the incidence angle for solar rays, ΔT is the difference of temperature according to the Equation (25), *K* is the dependency factor of $\eta_{opt,\;0}$ over θ as a result of Equation (26) and DNI is the normal incidence irradiance in W/m^2^:

(25) $$\Delta T=\frac{T_{SF,\;in}+T_{SF,\;out}}{2}-T_{amb}$$

Here $T_{SF,\;in}$ is the inlet temperature of heat transfer fluid in ℃, $T_{SF,\;out}$ is the outlet temperature of heat transfer fluid in ℃ y $T_{amb}$ is the ambiance temperature in ℃, and

(26) K=IAM·cosθ

where IAM is the incidence angle modifier according to Equation (12):

(27) $$IAM=1-\frac{a_{1}\cdot \theta +a_{2}\cdot \theta^{2}+a_{3}\cdot \theta^{3}}{\cos\theta }$$

The last equation $a_{i}$ contains empiric parameters from the manufacturer of the collector. The efficiency obtained for the parabolic trough collector can be seen in Figure 10b. In addition, although it has been said that the main goal of this device is not energy production (its target is monitoring meteorological data), it can be interesting to analyze the power consumption (in order of magnitude) of the linear actuator and stepper motor to make a comparison with the energy generated.

In relation to the linear actuator (a Firgelli model L12-100-100-6R) and its features according to its technical datasheet, the electrical working conditions of 450 mA at 6 V can be obtained, so considering Equation (28):

(28) P=u·i

It is easy to obtain the result of the power substracted to power generated, but only in moments when the linear actuator is working, that is, only when it is acting to extend/contract the solar collectors. It implies a consumption of 2.7 W. Making the hypothesis of a working time of 1 s per movement, and a change of position every 6 min, that implies 10 s/h of working time. Assuming 8 h/day of sun light duration as a medium value in Madrid, this adds up to 80 s/day. In such a way the final quantity of consumed energy is 216 J, according to Equation (29):

(29) E=P·t

On the other hand, following the same procedure and starting points, the electrical working conditions of the stepper (model 28BYJ48) are: 1 mA at 5 V. Considering again Equation (28), the power substracted is 0.05 W and the energy consumed, according to Equation (29) sums only 4 J.

## 3. Results

**Figure 11:**
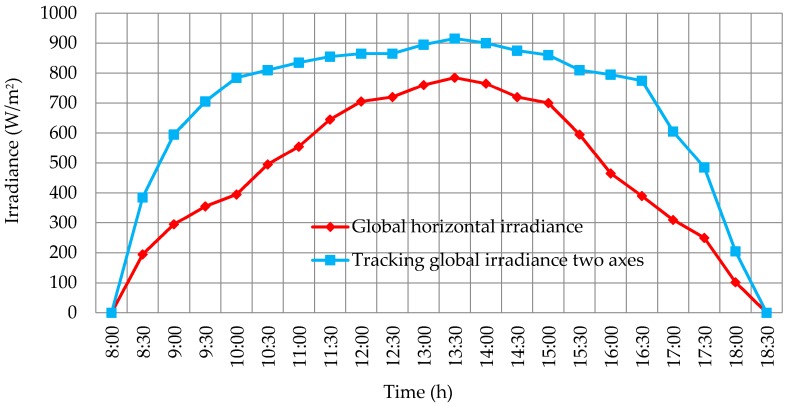
Averages values of irradiance every 30 min.

**Figure 12:**
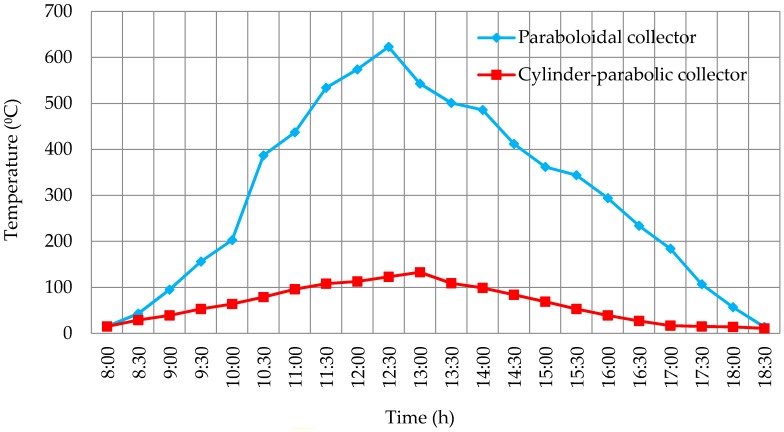
Collectors temperature every 30 min.

In the first place, measures of irradiance and temperature were taken with the help of the thermal solar tracker throughout winter in 2015 and 2016. The data were collected sequentially with the Arduino device every 30 min from 8.00 a.m. to 18.30 p.m., that is, from sunrise to sunset from December until March in Madrid, except for rainy days. The averages of irradiance obtained are shown in Figure 11. It can be seen how the correct calibration of the device reflects higher values at the times closer to midday. On the other hand, the temperature average for both sensors (paraboloidal and parabolic trough) is shown in Figure 12.

As it can be inferred from Figure 12, the temperature peaks are higher in the paraboloidal transducer, whose geometry and concentration ratio can reach values above the parabolic trough collector for the same conditions of irradiance. As a check, it was decided to carry out an inspection using thermal images that allow to verify the results obtained by the sensor.

**Figure 13:**
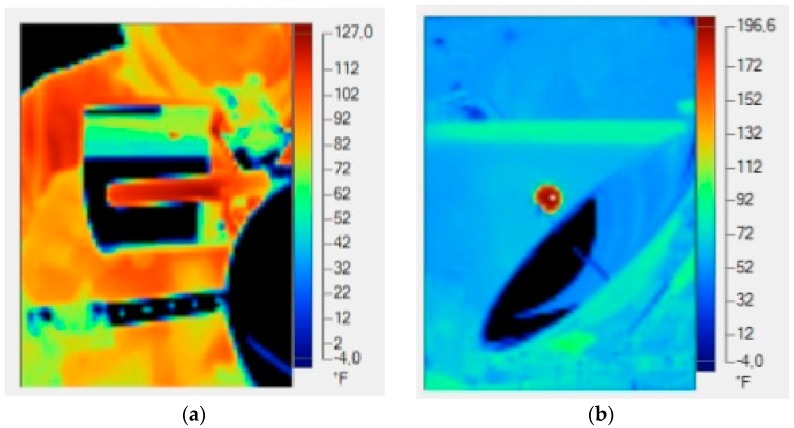
Inspection using thermographic camera. (**a**) Parabolic trough collector thermography; (**b**) Paraboloidal collector thermography.

**Figure 14:**
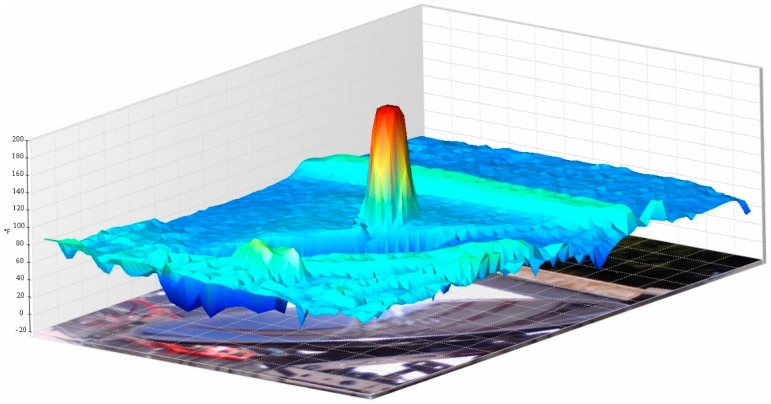
3D graph that shows Stirling dish collector temperatures.

**Figure 15:**
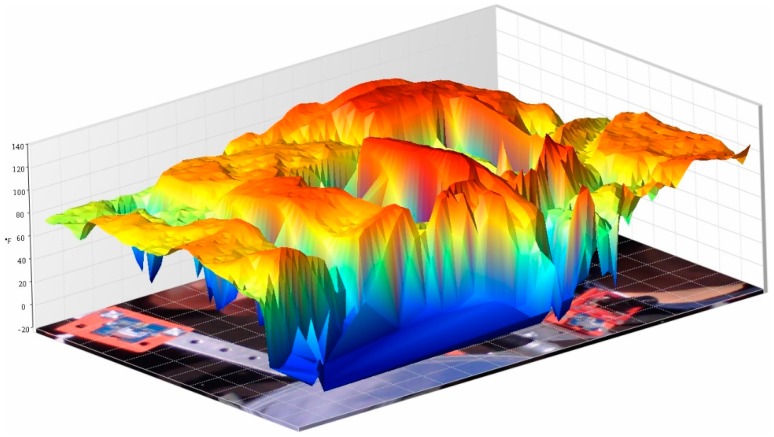
3D graph that shows the parabolic trough collector temperatures.

In this regard, two representative thermographic images (Figure 13) of both collectors taken at 12:00 p.m. and then proceeded to processing them using the software SmartView 3.12© were taken. In addition, the three-dimensional representation of the temperatures is displayed in function of the geometry of both collectors (Figure 14 and Figure 15), since it identifies the higher ones. Specifically, observing the paraboloidal collector (Figure 14) is appreciated, as expected, a very pronounced peak in its focus and in the parabolic trough a higher temperature along the cylindrical receiver (Figure 15) is also displayed.

## 4. Conclusions

In this paper it has been possible to design, model, build and calibrate a mechatronic paraboloidal and parabolic trough thermal solar tracker prototype which allows one to collect meteorological and energy data more reliably than using traditional methods based on satellite estimations obtained by the reflection of the incident radiation on the Earth. Furthermore, a scaled simulation of the behaviour of both collectors (paraboloidal and parabolic trough) has been achieved, anticipating the results regarding the energy potential in buildings wherever it takes place.

However, as further research lines and future implementation of this prototype in buildings, it would be appropiate to create a shell which can allow the degrees of freedom of the prototype movement and ensure the protection of the electronic components outdoors. In addition, it could be possible to use paraboloidal collectors whose geometry is less parabolic becoming pseudo-flat and reducing the visual impact, or retractable collectors which in case of a lack of irradiance could fold their surface up in a small sheet form favoring building integration.

## Figures and Tables

**Table 1:** Devices used in the construction of the prototype.

Terminology	Function
Arduino Mega 2560 ADK	Programmable electronic controller of the solar tracker, 256 Kb
Pololu Nema 17	Bipolar step by step motor 3.2 kg·cm torque and step angle 1.8°, to azimuthal tracking
Firgelli L12-100-100-6R	Linear actuator up to 17 mm/s and forces higher than 30 N for elevation tracking
SeedStudio	(a) Vibration piezoelectric sensor to measure the impact over the prototype of hail or heavy rain (b) Water detection sensor over the solar tracker (c) Ambiance temperature and relative air humidity sensor (d) Barometric pressure monitoring sensor (e) Quality air sensor in terms of CO_2_ concentration (f) Irradiance sensor in the four cardinal positions of the solar tracker, in W/m^2^ (g) Electronic compass installed in the brace head (h) Three axis accelerometer to identify movement and orientation of the solar tracker in order to follow its position (i) Digital camera to take pictures in real time
Adafruit Max6675	Temperature sensor (up to 500 °C) and signal amplifier
Todoelectrónica 6710 WIND02	Windmeter (speed of wind 10 km/h = 4 turns/s)
Ventus Ciencia and PHYWE	Paraboloidal and parabolic trough collectors, respectively

**Table 2:** Irradiance sensors.

Abbreviation	Sensor
NS	North Sensor, located in the upper part of the CSP solar tracker
SS	South Sensor, located in the lower part of the CSP solar tracker
ES	East Sensor, located in the right part of the CSP solar tracker
WS	West Sensor, located in the left part of the CSP solar tracker

## References

[1] Gil C.M., Gil M.-A.C., Castro M., Santos C.A., EIbañez C.J. (2001). Energía Solar Térmica de Media y Alta Temperatura (Monografías Técnicas de Energías Renovables).

[2] Imadojemu H.E. (1995). Concentrating parabolic collectors: A patent survey. Energy Convers. Manag..

[3] Feng C., Zheng H., Wang R., Ma X. (2016). Perfomance investigation of a concentrating photovoltaic/termal system with transmissive Fresnel solarconcentrator. Energy Convers. Manag..

[4] Ahmadi M.H., Sayyadi H., Dehghani S., Hosseinzade H. (2013). Designing a solar powered Stirling heat engine based on multiple criteria: Maximized thermal efficiency and power. Energy Convers. Manag..

[5] Arora R., Kaushik S.C., Kumar R., Arora R. (2016). Multi-objective thermo-economic optimization of solar parabolic dish Stirling heat engine with regenerative losses using NSGA-II and decision making. Electr. Power Energy Syst..

[6] Tlili I., Timoumi I., Nasrallah S.B. (2008). Thermodynamic analysis of the Stirling heat engine with regenerative losses and internal irreversible. Int. J. Engine Res..

[7] Aldegheri F., Baricordi S., Bernardoni P., Brocato M., Calabrese G., Guidi V., Mondardini L., Pozzetti L., Tonezzer M., Vincenzi D. (2014). Building integrated low concentration solar system for a self-sustainable Mediterranean villa: The Astonyshine house. Energy Build..

[8] Mao Q. (2016). Recent developments in geometrical configurations of termal energy storage for concentrating solar power plant. Renew. Sustain. Energy Rev..

[9] Liu M., Steven T., Bell S., Belusko M., Jacob R., Will G., Saman W., Bruno F. (2016). Review on concentrating solar power plants and new developmentes in high temperatura termal energy storage technologies. Renew. Sustain. Energy Rev..

[10] Lamnatou C., Mondol J.D., Chemisana D., Maurer C. (2015). Modelling and simulation of Building-Integrated solar thermal systems: Behaviour of the system. Renew. Sustain. Energy Rev..

[11] Bakos G.C. (2006). Design and construction of a two-axis Sun tracking system for parabolic trough collector (PTC) efficiency improvement. Renew. Energy.

[12] Skouri S., Ben Haj A., Bouadila S., Ben Salah M., Ben Nasrallah S. (2016). Design and construction of sun tracking systems for solar parabolic concentrator displacement. Renew. Sustain. Energy Rev..

[13] Chong K.K., Wong C.W. (2009). General formula for on-axis sun tracking system and its application in improving tracking accuracy of solar collector. Sol. Energy.

[14] Reda I., Andreas A. (2004). Solar position algorithm for solar radiation applications. Sol. Energy.

[15] Beltrán J.A., González J.L.S., García-Beltrán C.D. Design, manufacturing and performance test of a solar tracker made by an embedded control. Proceedings of the Electronics Engineering, Robotics and Automotive Mechanics Conference (CERMA 2007).

[16] Chen Y.T., Lim B.H., Lim C.S. (2006). General sun tracking formula for heliostats with arbitrarily oriented axes. J. Sol. Energy Eng..

[17] Falck D.Y., Colle`e B. (2012). Freecad [How-To].

[18] Warren J.-D., Adams J.Y., Molle H. (2001). Arduino Robotics.

[19] Salamone F., Belussi L., Danza L., Ghellere M.Y., Meroni I. (2015). An open source low-cost wireless control system for a forced circulation solar plant. Sensors.

